# Rapid Cas13a-based *pen*A genotyping for cefixime susceptibility in *Neisseria gonorrhoeae*

**DOI:** 10.1128/msphere.00182-26

**Published:** 2026-05-19

**Authors:** Thi Hai Yen Nguyen, Sakshi Garg, Gordon Adams, Sreekar Mantena, Nisha Gopal, Ho-Jun Suk, Jeffrey D. Klausner, Pardis C. Sabeti, Jacob E. Lemieux, Lao-Tzu Allan-Blitz

**Affiliations:** 1 Division of Global Health Equity, Brigham and Women's Hospital 1861, Boston, Massachusetts, USA; 2 Division of Infectious Diseases, Massachusetts General Hospital 2348, Boston, Massachusetts, USA; 3 Broad Institute of Massachusetts Institute of Technology and Harvard 33577, Boston, Massachusetts, USA; 4 DxLab Inc., Somerville, Massachusetts, USA; 5 Department of Population and Public Health Sciences, Keck School and Medicine, USC 12223 , Los Angeles, California, USA; 6 Department of Organismic and Evolutionary Biology, Harvard University 735342, Boston, Massachusetts, USA; 7 Department of Immunology and Infectious Disease, Harvard T.H. Chan School of Public Health 234189 , Boston, Massachusetts, USA; 8 Howard Hughes Medical Institute 2405, Chevy Chase, Maryland, USA; 9 Division of Infectious Diseases, Department of Medicine, University of California 229277, Los Angeles, California, USA; Medical College of Wisconsin, Milwaukee, Wisconsin, USA

**Keywords:** *Neisseria gonorrhoeae*, antimicrobial resistance, cefixime, CRISPR, Cas13, point-of-care, resistance-guided therapy

## Abstract

Antimicrobial resistance in *Neisseria gonorrhoeae* is an urgent public health threat. Resistance-guided therapy can assure appropriate treatment and reintroduce alternative therapeutic options by identifying genetic predictors of resistance. Mosaicism at codons 375–377 of the *pen*A gene is associated with cefixime resistance. Rapid, field-deployable assays for predicting cefixime susceptibility are lacking. We used a machine-learning algorithm to develop a CRISPR Cas13a-based assay to detect the absence of mosaicism at codons 375–377 of the *pen*A gene, combined with isothermal amplification in a single reaction. We integrated the assay onto a portable fluorescence-based platform. We evaluated performance using cultured isolates and compared results with PCR genotyping and phenotypic antimicrobial susceptibility testing. We also assessed the feasibility of reagent lyophilization of an existing *por*A *N. gonorrhoeae* detection assay, which can support cold-chain-independent deployment. Among 40 *N. gonorrhoeae* isolates, the Cas13a *pen*A assay demonstrated 100% concordance with PCR genotyping and 92.5% (95% confidence interval 79.6%–98.4%) concordance with phenotypic cefixime susceptibility. Median time to detection of the *pen*A mosaic assay was 12 min (interquartile range [IQR] 5 min). The lyophilized *por*A *N. gonorrhoeae* detection system detected all 12 isolates with a median time to detection of 45 min (IQR 40–45) compared to 45 min (IQR 35–50) for the positive aqueous control, although peak fluorescence was higher for the aqueous control (*P* < 0.01). The Cas13a assay was rapid and demonstrated strong correlation with genotypic and phenotypic cefixime susceptibility in *N. gonorrhoeae*, while a lyophilized assay retained functionality.

**IMPORTANCE:**

Antimicrobial resistance in *Neisseria gonorrhoeae* is an urgent threat to public health. Recent reports have highlighted the continued rise in ceftriaxone resistance, our last-line empiric treatment option. Molecular assays that detect the genetic determinants of resistance can improve antibiotic stewardship and increase the therapeutic options for cases of gonorrhea. As a result, those tests can reduce the pressure toward the emergence of ceftriaxone resistance. However, most molecular assays cannot be deployed in low-resource settings due to a lack of infrastructure. We report on the development of a point-of-care assay for predicting resistance to an oral first-line treatment option, cefixime. The assay provided results in under 30 min and demonstrated strong correlation with phenotypic and genotypic resistance. Furthermore, we report on the proof-of-concept freeze-drying of assay reagents, which could permit cold-chain-independent use. Collectively, our study provides the groundwork for developing and deploying such molecular resistance assays in low-resource settings.

## INTRODUCTION

Antimicrobial resistance in *Neisseria gonorrhoeae* infection is an urgent public health threat (1, 2). There were over 80 million new cases of gonorrhoeae worldwide in 2020 (3), with the highest prevalence among low-resource settings (4). Furthermore, *N. gonorrhoeae* has now developed resistance to all antimicrobials used in its treatment, including recent reports of high rates of resistance to ceftriaxone, the last-line empiric therapeutic option (5–7). Because routine culture is not feasible and because results would not be available rapidly enough to inform care, the diagnostic paradigm relies on nucleic acid amplification testing, which provides no information on antibiotic susceptibility. Thus, all *N. gonorrhoeae* infections are treated with a single regimen, which applies selective pressure toward the emergence of resistance (8). Furthermore, low-resource settings lack laboratory infrastructure to support nucleic acid amplification testing. Instead, such areas utilize syndromic management, which misses the high proportion of asymptomatic cases and results in antibiotic overuse among symptomatic patients (9), further driving resistance.

Rapid prediction of antimicrobial susceptibility to guide care, known as resistance-guided therapy, is possible via molecular assays that detect the genetic determinants of resistance (10). The absence of mutation in codon 91 of the gyrase A (*gyr*A) gene was more than 98% sensitive and 98% specific for predicting ciprofloxacin susceptibility (11*). Gyr*A genotyping assays are increasingly available using polymerase chain reaction (PCR), recombinant polymerase amplification (RPA), and even CRISPR (12–14). Resistance-guided therapy is increasingly being used for other pathogens as well, including *Staphylococcus aureus* and *Helicobacter pylori* (15, 16). The 2021 U.S. Centers for Disease Control and Prevention sexually transmitted infection treatment guidelines permit the use of ciprofloxacin in settings where rapid genotyping assays are available (17). However, the effectiveness of resistance-guided therapy to mitigate the selective pressure toward ceftriaxone resistance is enhanced when molecular detection platforms incorporate genetic targets that predict resistance to additional antibiotics (18).

Prior work has demonstrated that the absence of mutation at any of six loci in the *pen*A gene was between 95% and 99% sensitive for predicting susceptibility to cefixime, with the absence of mosaic insertions in codons 375–377 of particular importance (19, 20). The World Health Organization lists cefixime as an acceptable alternative first-line agent for uncomplicated gonorrhea (21); thus, rapid prediction of cefixime resistance may be able to avert treatment failures. We aimed to develop a low-cost, field-deployable system for rapidly determining the absence of *pen*A mosaicism in *N. gonorrhoeae*. To do so, we leveraged the Specific High-Sensitivity Enzymatic Reporter Unlocking (SHERLOCK) platform we previously utilized to develop assays for *N. gonorrhoeae* detection and *gyr*A genotype prediction (13, 22). That system employs isothermal amplification, T7-polymerase-mediated transcription, and RNA-guided CRISPR enzyme Cas13a detection via cleavage of a quenched reporter (23). We further aimed to utilize a field-deployable device for simultaneous amplification, transcriptionc, and detection. In addition, we aimed to lyophilize or freeze-dry reagents to permit cold-chain-independent storage and facilitate deployment in field settings.

## MATERIALS AND METHODS

### Cas13a guide RNA and RPA primer designs

To design Cas13a guide RNA (gRNA) sequences, we used a machine-learning tool known as Building Artificial Diagnostic Guides by Exploring Regions of Sequences (BADGERS) (24). BADGERS employs a predictive model of guide–target activity to explore a fitness landscape of candidate guide sequences and design those with optimal on-target and minimal off-target activity. As input, we provided 2,274 unique *N. gonorrhoeae* genomes (*n* = 1,675 with mosaicism in the *pen*A gene) and specified codons 375–377 as the design window. We provided nucleotide sequences of *N. gonorrhoeae* isolates with mosaicism at codons 375–377 in the *pen*A gene as the first target set and nucleotide sequences of *N. gonorrhoeae* isolates without mosaicism at those positions as the second target set. We selected potential candidate CRISPR gRNAs for the absence of *pen*A mosaicism based on scores generated by BADGERS for predicted fitness as well as predicted mean on- and off-target binding affinity while also attempting to ensure heterogeneity in the start codon position.

We then developed two forward and reverse RPA primer sets (P1 and P2) flanking the mosaic region using PrimerBlast (National Center for Biotechnology Information). We designed the primers to yield amplicons ranging from 140 to 200 base pairs in length, with melting temperature between 58°C and 68°C. To enable *in vitro* transcription, we appended a T7 RNA polymerase promoter sequence (5′-GAAATTAATACGACTCACTATAGG-3′) to the 5′ end of each forward primer. We evaluated each gRNA–primer pair in a one-pot SHERLOCK reaction (see below) to assess both amplification efficiency and discrimination between target and non-target sequences. We used synthetic *pen*A DNA that included the primer binding regions and gRNA binding site.

### SHERLOCK one-pot reaction

We performed the one-pot SHERLOCK reactions under standard Streamlined Highlighting of Infections to Navigate Epidemics (SHINE) conditions as previously described (25). Briefly, the master mix contained 1× SHINE buffer (20 mM HEPES, pH 8.0, with 60 mM KCl, and 5% polyethylene glycol [PEG]), 45 mM *Lwa*Cas13a (GenScript, Z03486-100), 1 U/µL murine RNase inhibitor (New England Biolabs, USA), 10 U/µL T7 RNA polymerase (Lucigen Corporation, USA), 136 mM RNaseAlert substrate v2 (Thermo Fisher Scientific, USA), and 2 mM of each ribonucleotide (rNTP) (New England Biolabs). Detailed information on reagents, including suppliers and stock concentrations, is provided in Table S1 and S2.

We used the prepared master mix to resuspend lyophilized TwistAmp Basic Kit RPA pellets (one pellet per 73.42 µL master mix volume), followed by magnesium acetate [Mg(OAc)_₂_] (TwistDx, United Kingdom) to a final concentration of 14 mM, which served as the sole magnesium cofactor. The mixture also included assay-specific concentrations of 320 nM of each forward and reverse RPA primers and 22.5 nM gRNA. We added target DNA to the final master mix at a 1:4 master mix-to-sample ratio. For evaluating gRNA and primer set performance, we ran the assays in triplicate on the Cytation 5 plate reader (Agilent Technologies, USA) at 37°C, measuring real-time fluorescence (excitation 485 nm, emission 528 nm) every 5 min for 3 h.

After selecting the gRNA and primer set, we evaluated the performance of the assay on cultured isolates using the DxHub platform (DxLab Inc., USA; manufactured under contract by Axxin, Australia). The DxHub is a portable, stand-alone platform that provides independent testing of up to eight individual tubes, with isothermal incubation between 38° and 72°C and dual-channel fluorescence detection in real time. We ran reactions for 60 min at 38°C and measured fluorescence kinetics every 20 s.

### Quantitative polymerase chain reaction

We designed a quantitative polymerase chain reaction (qPCR) system for confirmatory genotyping of the mosaic *pen*A region codons 375–377. We designed the assay such that the forward primer overlapped the non-mosaic consensus sequence; thus, any amplification would indicate the absence of mosaicism. The forward and reverse primer sequences targeting codons 375–377 of the *N. gonorrhoeae pen*A gene were 5′-GCTGAATACGCAGCCTTATAAAATCGG-3′ and 5′-TTTCTCAACAAACCTGCAGTTTCCC-3′, respectively.

We used 1× FastStart SYBR Green Master Mix (Sigma-Aldrich, USA), 0.5 µM of each primer, and DNA template in a 1:9 template-to-master mix ratio. We adjusted the final reaction volume to 10 µL with nuclease-free water and performed reactions in triplicate on a 384-well plate using a QuantStudio 6 System (Applied Biosystems, USA). We used the following thermal cycling conditions: initial denaturation at 95°C for 3 min, 40 cycles at 95°C for 15 s, 60°C for 1 min, and 72°C for 1 min, followed by a final extension at 68°C for 2 min. We measured fluorescence signals during the extension phase.

### Cas13a-based penA genotyping on *N. gonorrhoeae* isolates

We evaluated the selected Cas13a assay performance on purified *N. gonorrhoeae* isolates that had been previously collected through a prior research study (26) and as a part of laboratory validation. All isolates were stored at −80°C in Microbank vials (Pro-Lab Diagnostics, Canada), which contained cryoprotectant beads designed to preserve bacterial viability during long-term storage. We cultured *N. gonorrhoeae* isolates on Thayer–Martin agar at 37°C in 5% CO_2_ for 24–48 h. Antimicrobial susceptibility for all isolates had been phenotypically characterized by agar dilution as previously described (26). We extracted DNA from the isolates using the DNeasy Blood and Tissue Kit (Qiagen, Germany) in accordance with the manufacturer’s instructions.

We tested each isolate with the Cas13a *pen*A mosaic assay on the DxHub and qPCR. The DxHub has a programmable threshold that defines sample positivity via integrating fluorescence amplitude and the rate of rise of the fluorescence signal. Once a threshold has been established, the DxHub can automate assay interpretation. However, as we had not yet established a positivity threshold for the new assay;, we defined a positive fluorescence signal as a peak at least three standard deviations above the negative control. We defined the time at which peak fluorescence occurred as the point at which the slope of the fluorescence amplitude equaled 0. We defined the time to positivity as the time to 1/2 peak fluorescence (analogous to the maximal slope).

The Clinical and Laboratory Standards Institute defines cefixime phenotypic resistance as a minimum inhibitory concentration (MIC) of ≥0.25 µg/mL, with intermediate susceptibility as an MIC of 0.125 µg/mL (27). We considered isolates with an MIC of ≥0.125 µg/mL as non-susceptible for comparison purposes and compared the performance of Cas13a *pen*A mosaicism determination with (i) qPCR results and (ii) phenotypic susceptibility profiles.

### Reagent lyophilization

The lyophilizing process involves three sequential steps: freezing, primary drying, and secondary drying. In the first step, a frozen matrix is formed, in which water converts into ice crystals and solutes are concentrated. During primary drying, we removed the frozen water by sublimation under vacuum and low-temperature conditions, followed by the removal of the remaining unfrozen water as the final step (secondary drying).

We lyophilized CRISPR assay components following previously reported strategies (25). For lyophilization experiments, we used a Cas13a-based *N. gonorrhoeae* detection assay targeting the *por*A gene that had previously been validated (22). We prepared lyophilized pellets using a modified SHINE buffer, termed LYO buffer (50 mM HEPES, pH 8.0, with 12.5% [wt/vol] sucrose and 375 mM mannitol as cryoprotectants). The master mix contained (1× LYO buffer, 2 mM each rNTP mix, RPA pellet, 1 U/µL RNase inhibitor, 45 nM *Lwa*Cas13a, 1 U/µL T7 RNA polymerase, 0.0625 µM 6U-FAM reporter [FAM-UUUUUU-quencher], 22.5 nM gRNA, and 0.12 µM of each RPA primer mix).

We flash-froze aliquots of the master mix in liquid nitrogen and lyophilized them overnight in a FreeZone 70,040 4.5L Freeze Dryer (Labconco, USA) at −50°C and a vacuum pressure of <0.4 mbar. We stored the lyophilized pellets at −20°C for stability. We reconstituted the lyophilized pellets in resuspension buffer [60 mM KCl, 3.5% PEG 8000, 25 mM Mg(AOc)2, nuclease-free water].

For those experiments, we used extracted DNA from cultured isolates and performed fluorescence-based detection on the Cytation 5. We evaluated isolates in triplicate and analyzed the mean results across technical repeats. We then evaluated the median peak fluorescence and interquartile range (IQR) as well as time to detection (1/2 peak fluorescence) and IQR across isolates, comparing the lyophilized system to an aqueous control (a reaction prepared as described above on the same day the evaluation was performed).

### Data analysis

To compare mean differences in fluorescence measured on the Cytation 5, we subtracted the background signal from the 10 final readings and calculated the geometric mean of those values. We then used Student’s *t*-test with Welch’s correction, assuming unequal variances, to compare across samples. For non-parametric data, we used Wilcoxon’s signed-rank tests. We defined statistical significance as *P* < 0.05. We generated all figures using GraphPad Prism version 9.5.1 (GraphPad Software, USA).

## RESULTS

### Selection and validation of primer-guide RNA pair for Cas13a-based penA assay

BADGERS produced 80 potential gRNA sequences (Table S3). The four selected gRNAs had a median composite fitness score of −0.03 (IQR −0.04 to −0.02), a median composite on-target activity score of −0.70 (IQR −0.73 to −0.67), and a median composite off-target activity score of −3.16 (IQR −3.37 to −2.57).

**Fig 1:**
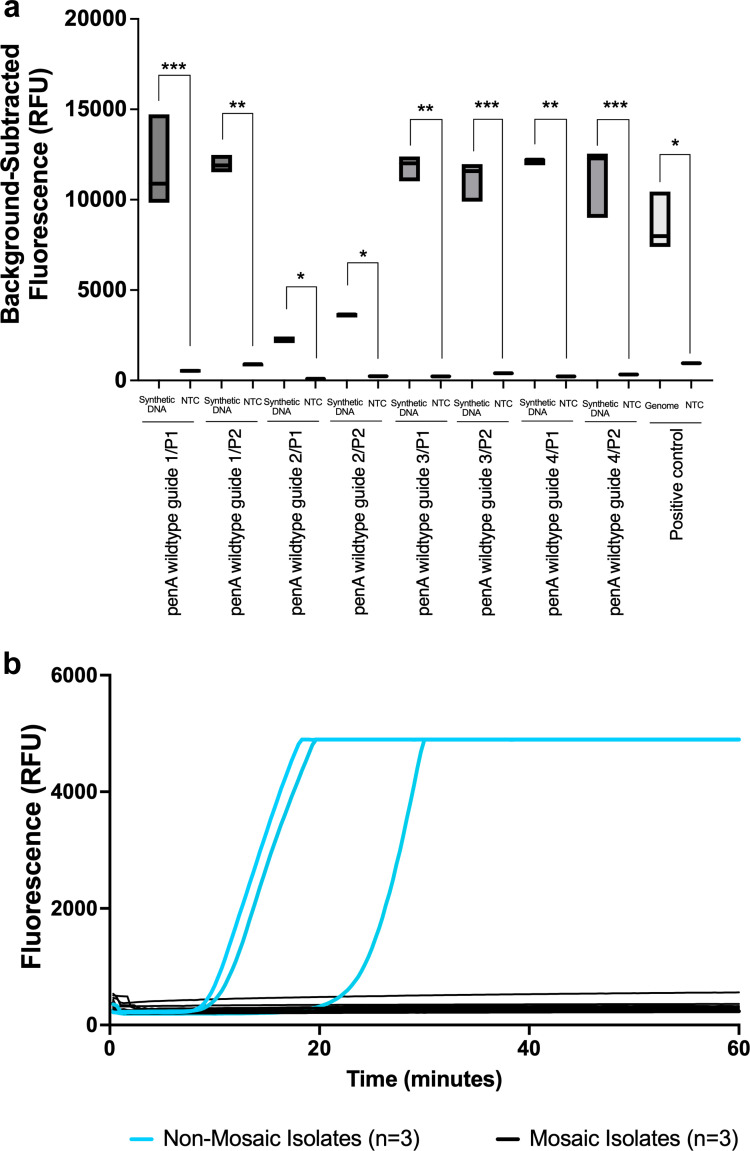
Performance of four gRNAs targeting codons 375–377 of the *pen*A gene, tested on a synthetic *N. gonorrhoeae* DNA with a positive control and a negative control (NTC) using a Cytation 5 (**a**). Cas13a-based *pen*A mosaic assay discriminated between non-mosaic (*n* = 3) and mosaic (*n* = 3) *N. gonorrhoeae* isolates using the gRNA 4–primer set 1 system on the DxHub (**b**). *P ≤ 0.01, **P ≤ 0.001, ***P ≤ 0.0001 (comparing geometric means).

**Fig 2:**
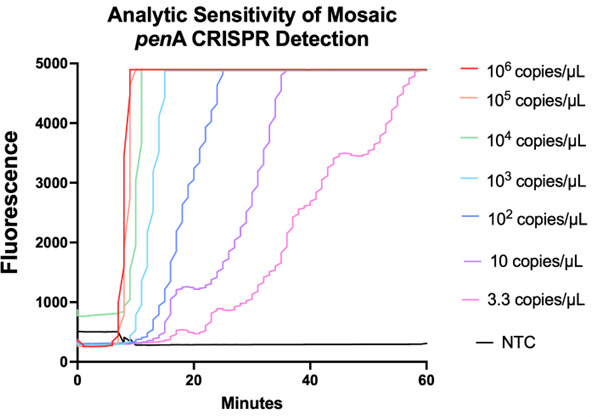
*In vitro* limit of detection of the Cas13a *N. gonorrhoeae pen*A assay on DxHub via 10-fold serial dilutions of a synthetic *pen*A DNA, and fluorescence signals were measured in real time.

The *pen*A non-mosaic gRNA 4 paired with primer set 1 produced the highest fluorescence signal and the greatest separation between the *pen*A target and negative controls (Fig. 1a). That assay correctly classified six *N. gonorrhoeae* isolates, including three non-mosaic and three mosaic strains (Fig. 1b). Furthermore, that assay detected non-mosaic synthetic DNA down to 3.3 copies/µL (Fig. 2). We therefore selected *pen*A non-mosaic gRNA 4 and primer set 1 for further analytical validation.

### Cas13a determination of non-mosaic penA genotypes among cultured isolates using the DxHub

**Fig 3:**
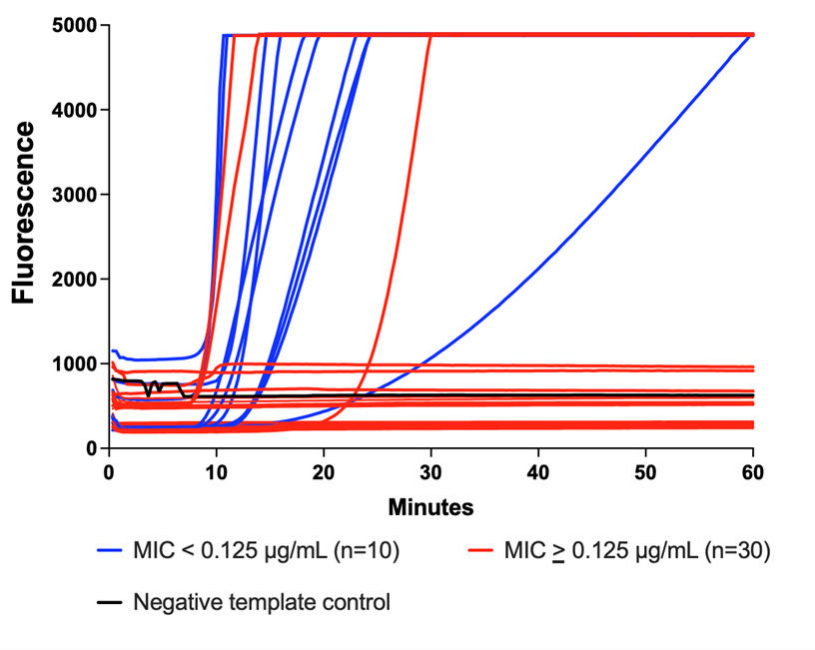
Performance of the Cas13a *pen*A assay for detecting cultured *N. gonorrhoeae* on a field-deployable platform. Blue lines represent cefixime-susceptible isolates (MIC < 0.125 µg/mL); red lines indicate cefixime non-susceptible isolates (MIC ≥ 0.125 µg/mL). Two isolates with MICs 0.25 µg/mL and one with MIC 0.125 µg/mL lacked *pen*A mosaicism. The black line represents the no-template control.

Of the 40 isolates included, 30 had MICs ≥0.125 µg/mL and 10 had MICs <0.125 µg/mL (Table 1). Among those isolates, the Cas13a *pen*A assay demonstrated 100% (95% confidence interval [CI] 91.2%–100%) concordance with qPCR. Three non-susceptible isolates (*n* = 2 with MIC of 0.25 µg/mL; *n* = 1 with MIC of 0.125 µg/mL) lacked the mosaic *pen*A allele, while no susceptible isolate harbored *pen*A mosaicism. The concordance of Cas13a-based *pen*A mosaic genotyping with phenotypic susceptibility was 92.5% (37/40 isolates; 95% CI 79.6%–98.4%). Figure 3 presents the diagnostic performance across all isolates. The median time to detection was 12 min (IQR 5 min).

**TABLE 1:** Phenotypic cefixime susceptibility profile of 40 *N. gonorrhoeae* isolates evaluated for *pen*A mosaicism using qPCR and a Cas13a-based assay

ID	Geographic region	Cefixime MIC (µg/mL)^*a*^	Resistance interpretation	*pen*A by qPCR	*pen*A by Cas13a^*b*^	Cas13a concordance	
						PCR	Phenotype
CCC001^*c*^	Hong Kong	0.25	Resistant	Non-mosaic	Non-mosaic	Yes	No
CCC018^*c*^	Hong Kong	0.25	Resistant	Non-mosaic	Non-mosaic	Yes	No
FQ009	United States	0.125	Intermediate	Non-mosaic	Non-mosaic	Yes	No
FQ023	United States	≤0.015	Susceptible	Non-mosaic	Non-mosaic	Yes	Yes
FQ027	United States	0.06	Susceptible	Non-mosaic	Non-mosaic	Yes	Yes
FQ030	United States	≤0.015	Susceptible	Non-mosaic	Non-mosaic	Yes	Yes
FQ038	United States	≤0.015	Susceptible	Non-mosaic	Non-mosaic	Yes	Yes
FQ043	United States	0.06	Susceptible	Non-mosaic	Non-mosaic	Yes	Yes
FQ047	United States	≤0.015	Susceptible	Non-mosaic	Non-mosaic	Yes	Yes
FQ049	United States	0.06	Susceptible	Non-mosaic	Non-mosaic	Yes	Yes
FQ056	United States	0.06	Susceptible	Non-mosaic	Non-mosaic	Yes	Yes
FQ059	United States	≤0.015	Susceptible	Non-mosaic	Non-mosaic	Yes	Yes
FQ093	United States	0.06	Susceptible	Non-mosaic	Non-mosaic	Yes	Yes
CCC006^*c*^	Hong Kong	0.25	Resistant	Mosaic	Mosaic	Yes	Yes
CCC009^*c*^	Hong Kong	2	Resistant	Mosaic	Mosaic	Yes	Yes
DDD004^*c*^	Hong Kong	2	Resistant	Mosaic	Mosaic	Yes	Yes
DDD021^*c*^	Hong Kong	0.5	Resistant	Mosaic	Mosaic	Yes	Yes
DDD022^*c*^	Hong Kong	1	Resistant	Mosaic	Mosaic	Yes	Yes
DDD035^*c*^	Hong Kong	0.5	Resistant	Mosaic	Mosaic	Yes	Yes
DDD044^*c*^	Hong Kong	1	Resistant	Mosaic	Mosaic	Yes	Yes
DDD050^*c*^	Hong Kong	0.5	Resistant	Mosaic	Mosaic	Yes	Yes
EEE004^*c*^	Hong Kong	2	Resistant	Mosaic	Mosaic	Yes	Yes
FQ091	United States	0.125	Intermediate	Mosaic	Mosaic	Yes	Yes
FQ041	United States	0.25	Resistant	Mosaic	Mosaic	Yes	Yes
FQ045	United States	0.25	Resistant	Mosaic	Mosaic	Yes	Yes
FQ048	United States	0.25	Resistant	Mosaic	Mosaic	Yes	Yes
FQ050	United States	0.25	Resistant	Mosaic	Mosaic	Yes	Yes
FQ053	United States	0.25	Resistant	Mosaic	Mosaic	Yes	Yes
FQ069	United States	0.25	Resistant	Mosaic	Mosaic	Yes	Yes
FQ071	United States	0.25	Resistant	Mosaic	Mosaic	Yes	Yes
FQ057	United States	0.25	Resistant	Mosaic	Mosaic	Yes	Yes
FQ060	United States	0.25	Resistant	Mosaic	Mosaic	Yes	Yes
FQ062	United States	0.25	Resistant	Mosaic	Mosaic	Yes	Yes
FQ074	United States	0.25	Resistant	Mosaic	Mosaic	Yes	Yes
FQ076	United States	0.25	Resistant	Mosaic	Mosaic	Yes	Yes
FQ078	United States	0.25	Resistant	Mosaic	Mosaic	Yes	Yes
FQ081	United States	0.25	Resistant	Mosaic	Mosaic	Yes	Yes
FQ083	United States	0.25	Resistant	Mosaic	Mosaic	Yes	Yes
FQ085	United States	0.25	Resistant	Mosaic	Mosaic	Yes	Yes
FQ088	United States	0.25	Resistant	Mosaic	Mosaic	Yes	Yes

^*a*^ Minimum inhibitory concentration.

^*b*^ Cas13a detection indicates the presence of a non-mosaic *penA* allele.

^*c*^ Isolates collected during a prior study (26).

### Cas13a-based *N. gonorrhoeae* detection using a lyophilized assay

**Fig 4:**
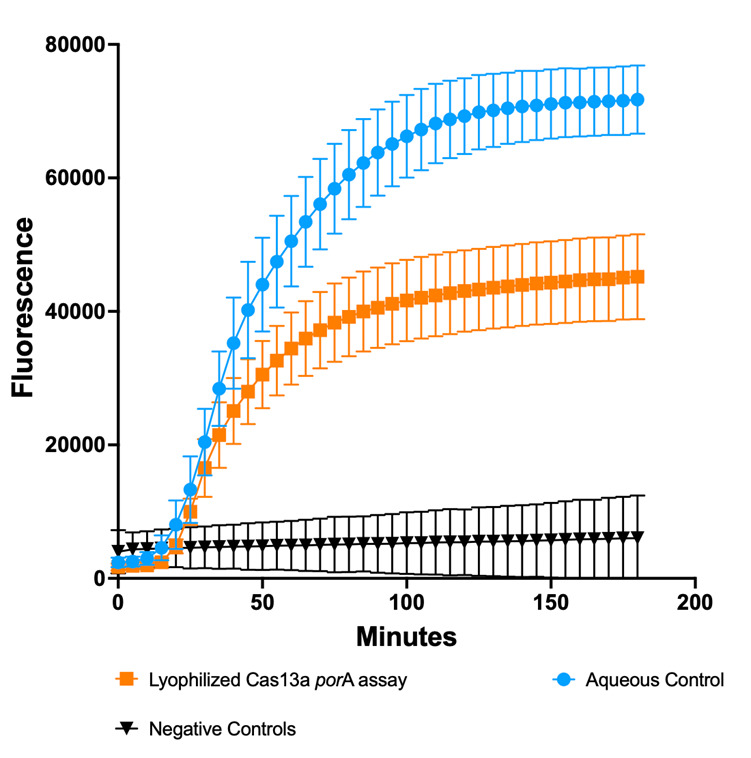
Fluorescence detection on the Cytation 5 fluorometer of 12 *N. gonorrhoeae* isolates evaluated in triplicate under aqueous conditions functioning as positive controls (blue lines), lyophilized pellets containing gRNA and primers (orange lines), and negative controls (black lines). The error bars represent the 95% confidence intervals. Significance was assessed using Wilcoxon signed-rank test.

As a proof of concept, we tested a total of 12 *N. gonorrhoeae* isolates using a lyophilized Cas13a system detecting the presence of the *por*A gene. The lyophilized system detected all 12 isolates, distinguishing those from negative controls (Fig. 4). The median time to detection for the lyophilized system was 45 min (IQR 40–45 min), while the median time to detection for the positive aqueous control was 45 min (IQR 35–55 min). The peak fluorescence amplitude was highest for the aqueous control (median 74,108 relative fluorescence units [RFUs]) compared with 46,802 RFUs for the lyophilized system (*P* < 0.01).

## DISCUSSION

This study aimed to develop a field-deployable, Cas13a-based method for rapidly determining the absence of mosaicism in the *pen*A gene of *N. gonorrhoeae*. The *pen*A mosaic assay accurately detected the absence of mosaicism compared with qPCR, which demonstrated promising correlation with phenotypic cefixime susceptibility. Integration of Cas13a assays into the DxHub may facilitate the use of such assays in field settings. Additionally, this study demonstrated promising proof-of-concept preservation of Cas13a-based *N. gonorrhoeae* detection system following lyophilization, which can permit cold-chain-independent storage and deployment of CRISPR assays in resource-limited settings.

While mosaic codons 375–377 are strongly associated with cefixime resistance, other mutations are also important predictors. Prior work developed a six-codon algorithm (positions 375–377, 501, 542, and 551) in *pen*A that predicted cefixime susceptibility in 99.5% of international isolates and 95.9% of additional strains in external data sets when no mutations were present (19, 20). Our results are consistent with those findings because 3 out of 40 isolates demonstrated genotypic and phenotypic discordance. As such, in its current form, use of this assay alone could misclassify a subset of non-susceptible isolates as susceptible. Therefore, it should be deployed in combination with detection of additional resistance targets or within a multi-target panel before being used to guide treatment decisions.

Another key substitution predictive of cefixime resistance is A501V/T (19, 20). One study recently reported the development of a CRISPR-Cas12a platform that detected both the mosaic *pen*A allele and mutations at codon 501, as well as genes for *N. gonorrhoeae* detection and a mutation associated with ceftriaxone resistance (28). That assay also represents a promising development toward field deployment of genetic resistance assays. However, Cas12a necessitates separating amplification from detection for diagnostics developed for DNA organisms due to the indiscriminate trans-cleavage activity of DNA by Cas12a (29). Conversely, Cas13a is an RNAse, which permits one-pot amplification and detection. Future work could aim to expand the repertoire of Cas13a tests to simplify and streamline workflow.

The benefits of rapid determination of cefixime susceptibility in *N. gonorrhoeae* are numerous. First, rapid molecular resistance assays may be able to guide appropriate use and reduce the risk of treatment failures. Second, while rapid molecular assays are increasingly available for predicting ciprofloxacin resistance, incorporation of rapid susceptibility prediction for additional classes of antibiotics may have a larger impact on delaying the emergence of ceftriaxone resistance (18). Furthermore, both cefixime and ciprofloxacin are oral therapies, which are often preferable for patients over intramuscular injection. Additionally, oral antibiotics can obviate the need to return to the clinic, potentially reducing losses to follow-up (30). Finally, prior work has demonstrated that the prevalence of *N. gonorrhoeae* infection with discordant genotypes between current sex partners is less than 3% (31); thus, among cefixime susceptible strains, such tests may be able to facilitate expeited partner therapy - an essential component of our public health strategy to mitigate the spread of STIs.

Our findings that lyophilized Cas13a reactions maintain functionality are also consistent with prior research (25). We did note a reduction in peak fluorescence and a potential slowing of reaction kinetics. Further optimization of lyophilized conditions may be able to improve the activity of the lyophilized assays; however, preservation of discriminatory capacity is a promising step toward cold-chain-free CRISPR assays. The development of low-cost, field-deployable resistance assays has the potential to extend the benefits of resistance-guided therapy to low-resource settings.

### Limitations

This study has several limitations. First, the sample size of *N. gonorrhoeae* isolates evaluated was small, which limits the precision of our findings. Additional evaluation among larger and geographically diverse samples is warranted. Similarly, future work will establish and validate a predefined positivity threshold using independent training and validation data sets, enabling prospective implementation with fully automated interpretation on the DxHub platform. Second, while *pen*A mosaicism is an important determinant of cefixime resistance, incorporation of additional single-nucleotide polymorphisms, such as those occurring at codons 501, 542, and 551, will be needed for comprehensive prediction of cefixime susceptibility. Furthermore, the assay is designed to detect the absence of *pen*A mosaicism, not specifically in *N. gonorrhoeae*; thus, it must be paired with similar assays that detect *N. gonorrhoeae* specifically to be useful clinically. Additionally, we used an in-house PCR assay as the reference comparator; further validation should use confirmatory sequencing. Finally, we assessed the stability and performance of lyophilized reagents under limited conditions using a previously developed *por*A assay. Further studies are needed to evaluate long-term storage and robustness across a range of temperatures and real-world field conditions, as well as to validate lyophilization for the *pen*A assay.

### Conclusion

We report on the development of a rapid, field-deployable Cas13a-based *pen*A mosaic assay that accurately detected the absence of mosaicism in the *pen*A gene of *N. gonorrhoeae*. The assay demonstrated promising correlation with phenotypic cefixime susceptibility. Integration into the portable DxHub platform could enable point-of-care use in field settings, while proof-of-concept lyophilization could support cold-chain-independent deployment, offering a practical tool for resistance-guided therapy and antimicrobial stewardship, particularly in resource-limited settings.

## SUPPLEMENTAL MATERIAL

Supplemental tables msphere.00182-26-s0001.docx Tables S1-S3.

ASM does not own the copyrights to Supplemental Material that may be linked to, or accessed through, an article. The authors have granted ASM a non-exclusive, world-wide license to publish the Supplemental Material files. Please contact the corresponding author directly for reuse.
