# Supplementary material for: Rapid Cas13a-based penA genotyping for cefixime susceptibility in Neisseria gonorrhoeae
Source: mSphere. 2026 May 19;11(6):e00182-26. doi: 10.1128/msphere.00182-26 (PMC13317185; doi:10.1128/msphere.00182-26)
Supplement: Supplemental tables — Tables S1-S3. [file msphere.00182-26-s0001.docx]

**Supplemental File**

**Supplemental Table 1: Reagents and stock concentrations used in the development of the Cas13a-based *Neisseria gonorrhoeae* and *pen*A mosaic assays, qPCR, DNA Sequencing.**

| Reagent | Reaction | Source | Stock Concentration | Notes |
| --- | --- | --- | --- | --- |
| C2c2 *Lwa*Cas13a | SHERLOCK | GenScript | 5 mg/mL | N/A |
| Rnase Inhibitor | SHERLOCK | NEB | 40 U/µL | Murine |
| T7 RNA Polymerase | SHERLOCK | Lucigen | 50 U/µL | NextGen |
| Reaction Buffer | SHERLOCK | N/A | 5X | 0·1 M HEPES pH 8·0; 300 mM KCl; 25% PEG-8000 |
| rNTPs | SHERLOCK | NEB | 25 mM of each nucleotide | N/A |
| RNase Alert Substrate v2 | SHERLOCK | Thermo Fisher Scientific | 2 µM | N/A |
| MgAc | SHERLOCK | TwistDx | 280 mM | TwistAmp Basic Kit |
| Storage Buffer | SHERLOCK | N/A | 1X | 50 mM Tris pH 7·5; 600 mM KCl; 5% glycerol; 2 mM DTT |
| RPA Primers  (forward, reverse) | SHERLOCK | Integrated DNA Technologies | 50 µM of each primer | Supplementary Table 2 for sequences |
| Cas13a gRNA (*pen*A) | SHERLOCK | Integrated DNA Technologies | 2·5 µM of each | Supplementary Table 2 for sequences |
| Nuclease-Free H2O |  | Thermo Fisher Scientific | N/A | Invitrogen |
| RPA Pellets (lyophilized) | SHERLOCK | TwistDx | N/A | TwistAmp Basic Kit |
| Synthetic *pen*A DNA (wildtype) | SHERLOCK | Integrated DNA Technologies | 10^10^ copies/µL | Supplementary Table 2 for sequences |
| PCR Primers (forward, reverse) | qPCR | Integrated DNA Technologies | 100 µM of each primer | Supplementary Table 2 for sequences |
| FastStart SYBR Green Master | qPCR | Roche | 2X | N/A |

**Supplementary Table 2: Primer, Guide, and Target sequences for qPCR and Cas13a-based *pen*A mosaic assays.**

| Reagent | Sequence |
| --- | --- |
| *Pen*A RPA Primer 1 (forward) | GAAATTAATACGACTCACTATAGGGCTGAATACGCAGCCTTATAAAATCGG |
| *Pen*A RPA Primer 1 (reverse) | TTTCTCAACAAACCTGCAGTTTCCC |
| *Pen*A RPA Primer 2 (forward) | GAAATTAATACGACTCACTATAGGGCATTATGCAGAAATCGTCCAACGTC |
| *Pen*A RPA Primer 2 (reverse) | AATTTCTCAACAAACCTGCAGTTTCCC |
| *Pen*A Cas 13a gRNA 1 | GAUUUAGACUACCCCAAAAACGAAGGGGACUAAAACCCGAAACGCGCAGACAGUUUGCUUGUGA |
| *Pen*A Cas 13a gRNA 2 | GAUUUAGACUACCCCAAAAACGAAGGGGACUAAAAGCGCCGAAACGCGCAGACAGUUUGCUUG |
| *Pen*A Cas 13a gRNA 3 | GAUUUAGACUACCCCAAAAACGAAGGGGACUAAAACCGCCGAAACGCGCAGACAGUUUGCUUGA |
| *Pen*A Cas 13a gRNA 4 | GAUUUAGACUACCCCAAAAACGAAGGGGACUAAAACCGCCGAAACGCGCAGACAGUUUGCUUGU |
| T7 promoter | GAAATTAATACGACTCACTATAGG |
| *pen*A qPCR Primer (forward) | GCTGAATACGCAGCCTTATAAAATCGG |
| *pen*A qPCR Primer (reverse) | TTTCTCAACAAACCTGCAGTTTCCC |

**Supplementary Table 3: Complete Output from BADGERS Containing Additional gRNA Candidates for Mosaic and Non-Mosaic *pen*A**

| **Machine-Learning Algorithm** | **Allele Target** | **Amino Acid Start Position** | **Guide Sequence** | **Fitness** | **Mean On-Target Activity** | **Mean Off-Target Activity** |
| --- | --- | --- | --- | --- | --- | --- |
| **WGAN** | WT375 | 976 | ACCGATTTGAACGAACGGCTGAATACGC | -0.0104 | -0.5837 | -3.5975 |
| **WGAN** | WT375 | 975 | AACCGATTTGAACGAACGGCTGAATACG | -0.0108 | -0.5955 | -3.7099 |
| **WGAN** | WT375 | 1389 | CAAAGAATCGACCGCGCGCGAGGTACGC | -0.0108 | -0.5898 | -3.4706 |
| **WGAN** | WT375 | 1512 | ACTGGTCAATGGCCGCTATGTGGACAAC | -0.0117 | -0.5828 | -3.2671 |
| **WGAN** | WT375 | 1528 | TCTGTGGACAACAAACACGTCGGTACGT | -0.0126 | -0.6222 | -3.0544 |
| **WGAN** | WT375 | 1096 | TTCGGCACAAGCAAACTGTCTGCGCGTT | -0.0130 | -0.6459 | -2.6775 |
| **WGAN** | WT375 | 1529 | ATGTGGACAACAAACACGTCGGTACGTT | -0.0134 | -0.6289 | -3.0967 |
| **WGAN** | WT375 | 1152 | TCATGAATTGGGCATCGGTGTGCGTATG | -0.0141 | -0.6680 | -2.8007 |
| **WGAN** | WT375 | 1408 | TAGGTACGCAATCTGATGGTTTCCGTAA | -0.0142 | -0.6226 | -3.1424 |
| **WGAN** | WT375 | 988 | CAACGGCTGAATACGCAGCCTTATAAAA | -0.0143 | -0.6662 | -3.2721 |
| **WGAN** | WT375 | 978 | CGATTTGAACGAACGGCTGAATACGCAG | -0.0145 | -0.6758 | -3.6372 |
| **WGAN** | WT375 | 954 | CAAGGCATTGGATGCGGGCAAAACCGAT | -0.0147 | -0.6282 | -2.7981 |
| **WGAN** | WT375 | 979 | TATTTGAACGAACGGCTGAATACGCAGC | -0.0147 | -0.6808 | -3.5922 |
| **WGAN** | WT375 | 980 | ATTTGAACGAACGGCTGAATACGCAGCC | -0.0149 | -0.6837 | -3.5329 |
| **WGAN** | WT375 | 1102 | ACAAGCAAACTGTCTGCGCGTTTCGGCG | -0.0152 | -0.6907 | -3.1758 |
| **WGAN** | WT375 | 1527 | CTATGTGGACAACAAACACGTCGGTACG | -0.0168 | -0.7046 | -3.0699 |
| **WGAN** | WT375 | 986 | ACGAACGGCTGAATACGCAGCCTTATAA | -0.0174 | -0.7262 | -3.5710 |
| **WGAN** | WT375 | 927 | CTCGGCAATCAAACCGTTCGTGATTGCG | -0.0175 | -0.7285 | -3.6130 |
| **WGAN** | WT375 | 1314 | ACTGACGCACGACGGCGTTTTGCTGCCG | -0.0175 | -0.7263 | -2.9948 |
| **WGAN** | WT375 | 1103 | CAAGCAAACTGTCTGCGCGTTTCGGCGC | -0.0177 | -0.7317 | -3.3687 |
| **WGAN** | MUT375 | 1096 | TTCGGTACCAGTAAACTTTCTGCCATGT | -0.0076 | -2.1529 | -3.9811 |
| **WGAN** | MUT375 | 1103 | CCAGTAAACTTTCTGCCATGTTTACGCC | -0.0088 | -2.2661 | -4.0008 |
| **WGAN** | MUT375 | 926 | TTTCTGCCATGAAGCCGTTTACCATTGC | -0.0100 | -1.5134 | -3.9908 |
| **WGAN** | MUT375 | 925 | TGTTCTGCCATGAAGCCGTTTACCATTG | -0.0104 | -1.5877 | -3.9984 |
| **WGAN** | MUT375 | 980 | ATGCAACCGATACATTCAATACCCTGCC | -0.0112 | -2.0369 | -4.0063 |
| **WGAN** | MUT375 | 1102 | ACCAGTAAACTTTCTGCCATGTTTACGC | -0.0123 | -2.3136 | -4.0008 |
| **WGAN** | MUT375 | 985 | ACCGATACATTCAATACCCTGCCTTACA | -0.0126 | -1.7214 | -4.0092 |
| **WGAN** | MUT375 | 1153 | CACGATTTAGGTGTGGGCGTGCGCATGC | -0.0136 | -1.9937 | -3.9282 |
| **WGAN** | MUT375 | 1101 | TCCCAGTAAACTTTCTGCCATGTTTACG | -0.0138 | -2.3313 | -4.0042 |
| **WGAN** | MUT375 | 1226 | TCAGATGGCAAAAAATCGAACAGGCAAC | -0.0141 | -3.0959 | -3.9344 |
| **WGAN** | MUT375 | 972 | CAAAGTGGATGCAACCGATACATTCAAT | -0.0143 | -2.0289 | -4.0014 |
| **WGAN** | MUT375 | 1152 | TCACGATTTAGGTGTGGGCGTGCGCATG | -0.0147 | -2.0236 | -3.8774 |
| **WGAN** | MUT375 | 957 | AGCATTGGATTCCGGCAAAGTGGATGCA | -0.0149 | -1.9516 | -4.0000 |
| **WGAN** | MUT375 | 1414 | CGCGAGTTGATGGTTTCCGTTACTGAAG | -0.0154 | -1.4608 | -3.8942 |
| **WGAN** | MUT375 | 1527 | TTACGTCGATTACAAACACGTTGCCACT | -0.0155 | -1.7315 | -3.5919 |
| **WGAN** | MUT375 | 984 | AACCGATACATTCAATACCCTGCCTTAC | -0.0155 | -1.6875 | -4.0142 |
| **WGAN** | MUT375 | 1124 | TTACGCCTAAAGAAATGTACGATTTCTA | -0.0167 | -2.4446 | -3.6817 |
| **WGAN** | MUT375 | 1097 | TCGGTACCAGTAAACTTTCTGCCATGTT | -0.0168 | -2.3521 | -4.0071 |
| **WGAN** | MUT375 | 975 | AGTGGATGCAACCGATACATTCAATACC | -0.0169 | -2.0789 | -4.0049 |
| **WGAN** | MUT375 | 971 | GCAAAGTGGATGCAACCGATACATTCAA | -0.0172 | -1.9508 | -4.0021 |
| **Evolutionary** | WT375 | 975 | TCCCGATTTGAACGAACGGCTGAATACG | -0.0259 | -0.5590 | -3.6605 |
| **Evolutionary** | WT375 | 976 | TCCGATTTGAACGAACGGCTGAATACGC | -0.0271 | -0.5787 | -3.5891 |
| **Evolutionary** | WT375 | 1389 | TAAAGAATCGACCGCGCGCGAGGTACGC | -0.0288 | -0.5872 | -3.4835 |
| **Evolutionary** | WT375 | 1096 | TTCGGCACAAGCAAACTGTCTGCGCGTT | -0.0319 | -0.6459 | -2.6775 |
| **Evolutionary** | WT375 | 1512 | GCTGGTCAATGGCCGCTATGTGGACAAC | -0.0324 | -0.5978 | -3.3071 |
| **Evolutionary** | WT375 | 1528 | CATGTGGACAACAAACACGTCGGTACGC | -0.0329 | -0.6220 | -3.0588 |
| **Evolutionary** | WT375 | 980 | TTTTGAACGAACGGCTGAATACGCAGCC | -0.0332 | -0.6619 | -3.5119 |
| **Evolutionary** | WT375 | 1152 | CCATGAATTGGGCATCGGTGTGCGTATG | -0.0335 | -0.6583 | -2.7802 |
| **Evolutionary** | WT375 | 1102 | TCAAGCAAACTGTCTGCGCGTTTCGGCG | -0.0337 | -0.6695 | -3.1406 |
| **Evolutionary** | WT375 | 979 | TCTTTGAACGAACGGCTGAATACGCAGC | -0.0339 | -0.6713 | -3.5730 |
| **Evolutionary** | WT375 | 978 | CCATTTGAACGAACGGCTGAATACGCAG | -0.0339 | -0.6707 | -3.6040 |
| **Evolutionary** | WT375 | 988 | TGACGGCTGAATACGCAGCCTTATAAAA | -0.0346 | -0.6615 | -3.2837 |
| **Evolutionary** | WT375 | 1529 | ACGTGGACAACAAACACGTCGGTACGTT | -0.0353 | -0.6177 | -3.0565 |
| **Evolutionary** | WT375 | 1100 | TCACAAGCAAACTGTCTGCGCGTTTCGG | -0.0376 | -0.7129 | -2.5727 |
| **Evolutionary** | WT375 | 1527 | TTATGTGGACAACAAACACGTCGGTACG | -0.0377 | -0.6863 | -3.1282 |
| **Evolutionary** | WT375 | 986 | TCGAACGGCTGAATACGCAGCCTTATAA | -0.0379 | -0.7153 | -3.5656 |
| **Evolutionary** | WT375 | 1314 | TCTGACGCACGACGGCGTTTTGCTGCCG | -0.0385 | -0.7117 | -2.9624 |
| **Evolutionary** | WT375 | 1103 | TCAGCAAACTGTCTGCGCGTTTCGGCGC | -0.0387 | -0.7250 | -3.3235 |
| **Evolutionary** | WT375 | 927 | TCCGGCAATCAAACCGTTCGTGATTGCG | -0.0388 | -0.7266 | -3.6127 |
| **Evolutionary** | WT375 | 1366 | TCGCCGCAAGGCAAACGCATATTCAAAG | -0.0390 | -0.7116 | -3.2370 |
| **Evolutionary** | MUT375 | 1096 | TTCGGTACCAGTAAACTTTCTGCCATGT | -0.0225 | -2.1529 | -3.9811 |
| **Evolutionary** | MUT375 | 1103 | TCAGTAAACTTTCTGCCATGTTTACGCC | -0.0240 | -2.2604 | -4.0009 |
| **Evolutionary** | MUT375 | 926 | TTTCTGCCATGAAGCCGTTTACCATTGC | -0.0269 | -1.5134 | -3.9908 |
| **Evolutionary** | MUT375 | 925 | TCTTCTGCCATGAAGCCGTTTACCATTG | -0.0270 | -1.5670 | -3.9974 |
| **Evolutionary** | MUT375 | 980 | TCGCAACCGATACATTCAATACCCTGCC | -0.0274 | -2.0085 | -4.0088 |
| **Evolutionary** | MUT375 | 1102 | TCCAGTAAACTTTCTGCCATGTTTACGC | -0.0296 | -2.3055 | -4.0009 |
| **Evolutionary** | MUT375 | 985 | TCCGATACATTCAATACCCTGCCTTACA | -0.0305 | -1.7070 | -4.0098 |
| **Evolutionary** | MUT375 | 984 | TCCCGATACATTCAATACCCTGCCTTAC | -0.0312 | -1.6382 | -4.0175 |
| **Evolutionary** | MUT375 | 1153 | TCCGATTTAGGTGTGGGCGTGCGCATGC | -0.0325 | -1.9492 | -3.8961 |
| **Evolutionary** | MUT375 | 1101 | CACCAGTAAACTTTCTGCCATGTTTACG | -0.0330 | -2.3303 | -4.0029 |
| **Evolutionary** | MUT375 | 972 | TCAAGTGGATGCAACCGATACATTCAAT | -0.0333 | -2.0087 | -4.0020 |
| **Evolutionary** | MUT375 | 957 | TGCATTGGATTCCGGCAAAGTGGATGCA | -0.0335 | -1.9327 | -3.9994 |
| **Evolutionary** | MUT375 | 971 | TCAAAGTGGATGCAACCGATACATTCAA | -0.0341 | -1.9031 | -4.0029 |
| **Evolutionary** | MUT375 | 1226 | TCAGATGGCAAAAAATCGAACAGGCAAC | -0.0343 | -3.0959 | -3.9344 |
| **Evolutionary** | MUT375 | 1414 | TCCGAGTTGATGGTTTCCGTTACTGAAG | -0.0353 | -1.4097 | -3.8538 |
| **Evolutionary** | MUT375 | 1152 | TCACGATTTAGGTGTGGGCGTGCGCATG | -0.0358 | -2.0236 | -3.8774 |
| **Evolutionary** | MUT375 | 975 | TGCGGATGCAACCGATACATTCAATACC | -0.0359 | -2.0554 | -4.0054 |
| **Evolutionary** | MUT375 | 979 | TCTGCAACCGATACATTCAATACCCTGC | -0.0368 | -2.0566 | -4.0035 |
| **Evolutionary** | MUT375 | 1097 | CCGGTACCAGTAAACTTTCTGCCATGTT | -0.0373 | -2.3483 | -4.0088 |
| **Evolutionary** | MUT375 | 981 | TCCAACCGATACATTCAATACCCTGCCT | -0.0382 | -2.0604 | -4.0149 |
| Red color indicates the selected guide sequences | | | | | | |
